# l-Glutamine Attenuates Apoptosis Induced by Endoplasmic Reticulum Stress by Activating the IRE1α-XBP1 Axis in IPEC-J2: A Novel Mechanism of l-Glutamine in Promoting Intestinal Health

**DOI:** 10.3390/ijms18122617

**Published:** 2017-12-05

**Authors:** Qian Jiang, Jiashun Chen, Shaojuan Liu, Gang Liu, Kang Yao, Yulong Yin

**Affiliations:** 1 Key Laboratory of Agroecological Processes in Subtropical Region, Institute of Subtropical Agriculture, Chinese Academy of Sciences, National Engineering Laboratory for Pollution Control and Waste Utilization in Livestock and Poultry Production, Changsha 410125, China; jiangqianisa@gmail.com (Q.J.); chenjiashun1988@163.com (J.C.); liushaojuan15@mails.ucas.ac.cn (S.L.); yinyulong@isa.ac.cn (Y.Y.); 2 University of the Chinese Academy of Sciences, Beijing 10008, China; 3 College of Animal Science and Technology, Hunan Agricultural University, Changsha 410125, China; 4 Hunan Co-Innovation Center of Animal Production Safety, CICAPS, Changsha 410128, China

**Keywords:** endoplasmic reticulum stress, IRE1α-XBP1, l-glutamine, intestinal porcine epithelial cell line J2

## Abstract

Intestinal absorption and barrier malfunctions are associated with endoplasmic reticulum stress (ERS) in the intestine. We induced ERS by exposing the intestinal porcine epithelial cell line J2 (IPEC-J2) to tunicamycin (TUNI) to explore the potential of l-glutamine to reduce ERS-induced apoptosis. Our experiments demonstrated that exposing cells to TUNI results in spontaneous ERS and encourages the upregulation of glucose-regulated protein 78 (GRP78). Prolonged TUNI-induced ERS was found to increase apoptosis mediated by C/enhancer binding protein homologous protein (CHOP), accompanied by GRP78 downregulation. Treatment with l-glutamine was found to promote cell proliferation within the growth medium but to have little effect in basic Dulbecco’s modified Eagle medium. Finally, in the milieu of TUNI-induced ERS, l-glutamine was found to maintain a high level of GRP78, alleviate CHOP-mediated apoptosis and activate the inositol requiring enzyme 1α (IRE1α)-X-box binding protein 1 (XBP1) axis. A specific inhibitor of the IRE1α-XBP1 axis reversed the protective effect of l-glutamine by blocking the expression of IRE1α/XBP1s. We propose that the functional effect of l-glutamine on intestinal health may be partly due to its modulation of ERS and CHOP-mediated apoptosis.

## 1. Introduction

The amino acid l-glutamine is considered to be the carbon source for purine and pyrimidine synthesis. It participates in the Coriolis cycle through deamination and related procedures [1], and becomes essential when the body suffers from metabolic stresses such as trauma [2], cancer [3], sepsis [4], or burns [5]. It is vital to ensure the intake of l-glutamine to meet the increased physiological needs arising from these conditions. Specifically, l-glutamine has received considerable attention in animal research for its health-promoting properties. For example, a supplement of l-glutamine administered to weaning piglets modified immune cells in the mesenteric lymph nodes and supported a T helper 1 type cytokine response after T cell stimulation [6]. In vitro, l-glutamine deficiency disturbs amino acid metabolism in intestinal epithelial cells [7], attenuates their mammalian target of rapamycin (mTOR) pathway [8] and downregulates mitogen-activated protein kinase/extracellular signal-regulated kinase signalling, thereby inhibiting protein synthesis and cell proliferation [9].

The endoplasmic reticulum (ER) is responsible for polypeptide synthesis, post-translational modification and folding to form proteins for cellular functions or secretion [10]. ER stress (ERS) is caused by the excessive accumulation of misfolded and unfolded proteins in the intestinal organs during protein biosynthesis [11]. Intestinal absorption and barrier malfunctions have been attributed to ERS [12,13,14]. Such stress triggers a series of signalling and transcriptional events known as the unfolded protein response (UPR) [15]. The enhanced UPR is triggered to maintain ER homeostasis in cells; its failure can lead to local inflammation in stressed cells and programmed cell death via apoptosis [16] or autophagy [17]. UPR activation of the IRE1α-XBP1 branch has shown to be involved in cell homeostasis and survival [18]. Similarly, research [19] has indicated that l-glutamine treatment attenuates ERS and apoptosis in 2,4,6-trinitrobenzenesulfonic acid (TNBS) induced colitis in rats. Compared with rats, pigs experience fiercer ERS as a consequence of pathogen infection [20] and digestive difficulties [21]. Although the health-promoting properties of l-glutamine in porcine intestine have been acknowledged, the molecular mechanisms underlying the regulation of ERS by l-glutamine are still poorly understood. 

The intestinal porcine epithelial cell line J2 (IPEC-J2), originally isolated from the jejunal epithelium of a neonatal unsuckled piglet [22], offers a realistic and representative means of mimicking porcine small intestine and provides a useful cell model for pharmacology research as well as studies of toxicity [23], microbiology [24], bioavailability [25], and metabolism [26] in the fields of veterinary medicine and animal science. 

In the study reported here, we selected IPEC-J2 as the cell model to cast new light on the role of l-glutamine in responding to ERS, and its molecular mechanism.

## 2. Results

### 2.1. Role of Tunicamycin (TUNI) in Spontaneous Endoplasmic Reticulum Stress (ERS)

**Figure 1:**
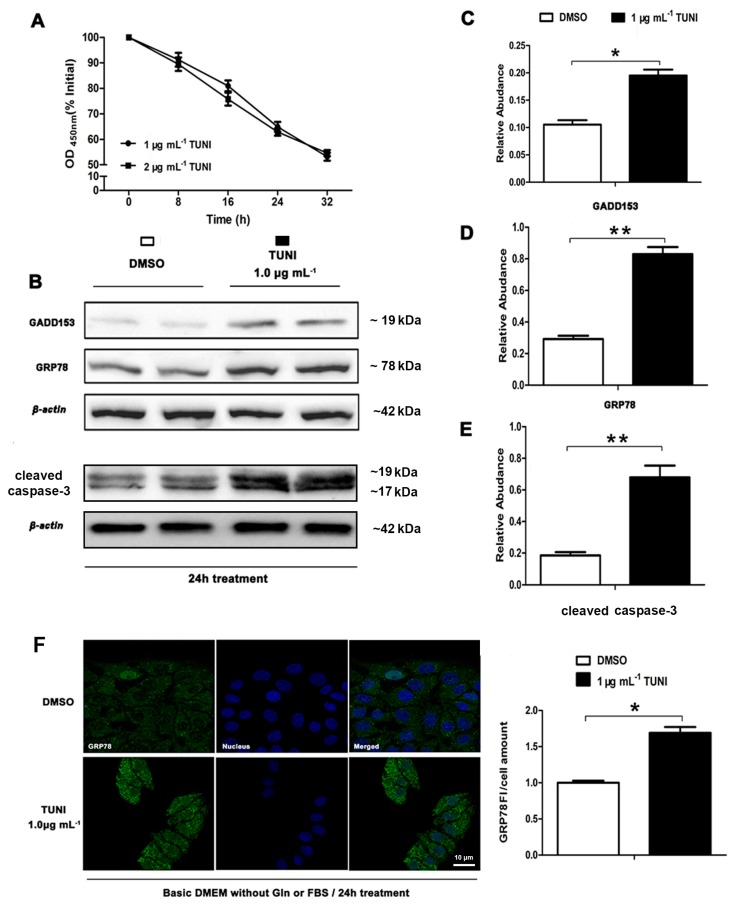
TUNI-induced (tunicamycin) endoplasmic reticulum stress (ERS) in intestinal porcine epithelial cell line J2 (IPEC-J2). (**A**) Cell viability assay performed using Cell Counting Kit 8 (CCK-8) after cells had been treated with 1.0 μg mL^−1^ TUNI for 0 to 32 h; (**B**) western blot analysis of ERS mark proteins after ERS induction; two representative protein bands from the three independent experiments are shown; (**C**,**D**) relative abundance of GRP78 and GADD153; (**E**) fluorescence staining of GRP78 in cells treated with dimethyl sulphoxide (DMSO) or TUNI (1 μg mL^−1^) for 24 h. Data given as means ± standard deviations (SDs) for three independent experiments (* means under *T* test, *p* < 0.05, ** means under *T* test, *p* < 0.01).

The UPR pathway is regularly triggered by ERS in the intestinal epithelium, and several reports have suggested that inflammatory bowel disease is involved in the induction of ERS. Specifically, activating transcription factor 6 (ATF6), ATF4 and spliced XBP-1 (XBP-1s) can regulate the expression of ER chaperone proteins that enhance ER folding ability, including glucose-regulated protein 78 (GRP78) and other stress genes, such as C/enhancer binding protein homologous protein (GADD153/CHOP). In the study reported here, we first used the TUNI-induced ERS model with IPEC-J2 to examine the potential role of TUNI in ERS signalling and ERS-mediated apoptosis. IPEC-J2 was treated with 1 μg mL^−1^ TUNI for 0, 8, 16, 24, or 48 h, and cell viability was assessed using the Cell Counting Kit 8 (CCK-8), as shown in Figure 1A. Based on our pre-experiments, 24 h treatment with 1 μg mL^−1^ TUNI was selected as the prolonged ERS cell model. Immunofluorescence analysis was performed to detect GRP78 levels (Figure 1F). The expression of GRP78, cleaved caspase-3 and GADD153/CHOP was evaluated using the Western blot (Figure 1B). As shown, TUNI was found to encourage the upregulation of GRP78 (Figure 1D,F) and increase the expression of GADD153/CHOP (Figure 1C) and cleaved caspase-3 (Figure 1E), indicating that spontaneous ERS and ERS-mediated cell apoptosis had been triggered in this cell model.

### 2.2. l-Glutamine Promotes Cell Proliferation in Normal Growth Medium

**Figure 2:**
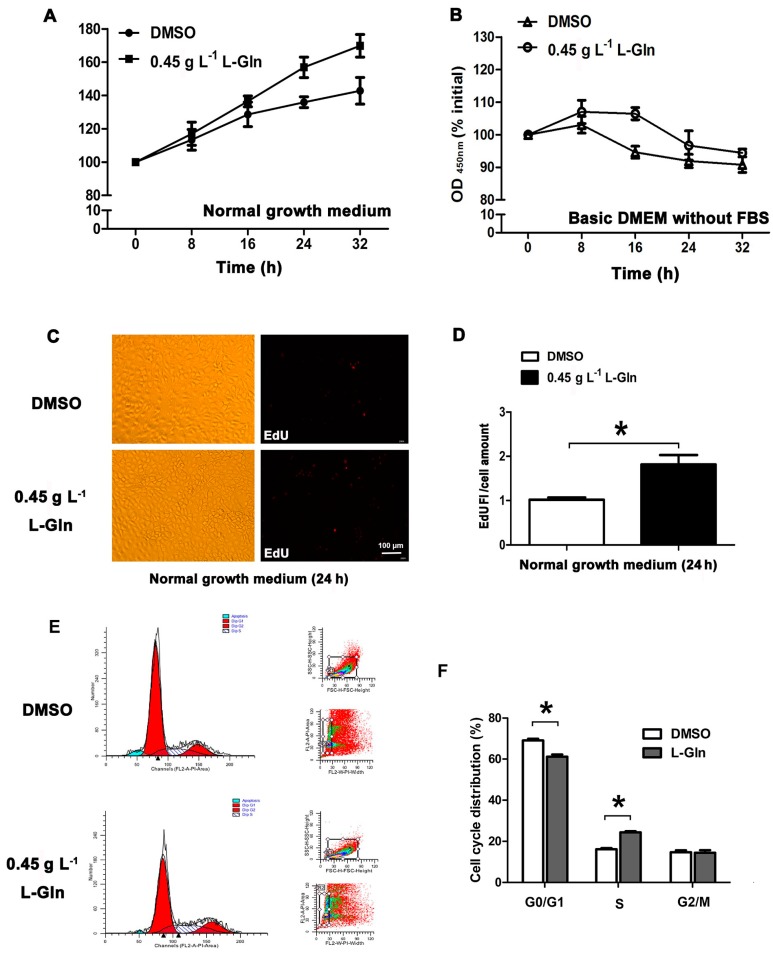
l-glutamine promotes cell proliferation within specific milieu. (**A**) In the normal growth medium, cells were treated with DMSO or 0.45 g L^−1^ l-glutamine for 0 to 32 h, followed by a cell viability assay using CCK-8; (**B**) In basic DMEM, relative cell viability was measured using CCK-8 after treating cells with DMSO or 0.45 g L^−1^ l-glutamine for 0 to 32 h; (**C**,**D**) After the treatment described in A, cell proliferation rate was determined by EdU staining, and the results are shown as relative EdU fluorescent light/cell values; (**E**,**F**) After the treatment described in A, cell cycle distribution was determined by flow cytometry; the statistical results are shown here. Data given as means ± SDs for the three independent experiments (* means under *T* test, *p* < 0.05, ** means under *T* test, *p* < 0.01).

To clarify the sole effect of l-glutamine on cell survival, we treated IPEC-J2 with l-glutamine in a normal growth medium (dulbecco’s modified eagle medium with 10% foetal bovine serum, FBS) and basic dulbecco’s modified eagle medium without 10% FBS), respectively. In the normal growth medium, cell cycle distribution was determined by flow cytometry (Figure 2E), and proliferation was observed by 5-ethynyl-2′-deoxyuridine (EdU) staining (Figure 2C). In the normal growth medium, l-glutamine treatment was found to improve cell viability (Figure 2A,D) and promote cell proliferation, as indicated by enhanced DNA replication (Figure 2C) and an increased number of cells in the S-phase (Figure 2E,F). However, in the basic DMEM, l-glutamine improved cell viability only for the first 16 h; this tendency receded over the following 16 h (Figure 2B).

### 2.3. l-Glutamine Reduces Apoptosis and Maintains a High Level of GRP78 in Response to ERS

**Figure 3:**
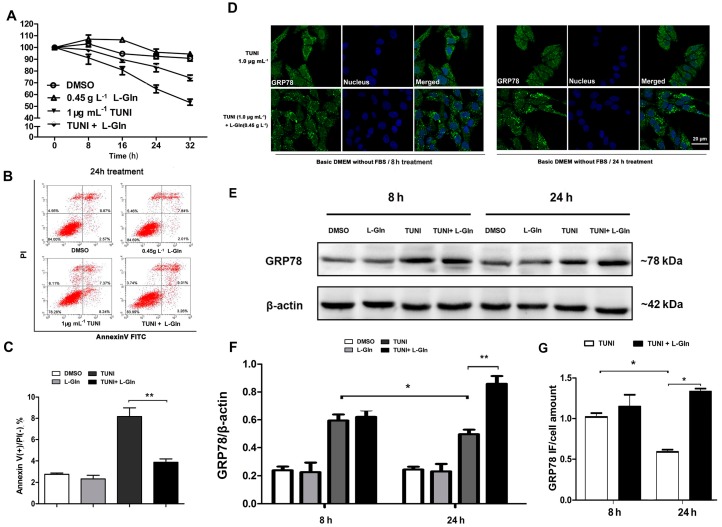
l-glutamine protects cell from TUNI-induced apoptosis. In basic DMEM, cells were treated with DMSO, 0.45 g L^−1^ l-glutamine and 1.0 μg mL^−1^ TUNI or a combination of l-glutamine and TUNI for 0 to 32 h. (**A**) Relative cell viability was measured using CCK-8; (**B**) PI/Annexin V-FITC staining and cell fluorescent screening were performed by flow cytometry; (**C**) Statistical results of Annexin V(+)/PI(−) staining; (**D**) GRP78 and nucleuses of cells in 8 and 24 h treatments were fluorescence labelled with Alexa Fluor 488 and DAPI, and excitation fluorescence was captured by laser scanning confocal microscopy; (**E**) Western blot analysis of GRP78 protein after ERS induction for 8 or 24 h; one representative protein band from the three independent experiments is shown; (**F**) Relative abundance of GRP78; (**G**) Relative fluorescence intensity of GRP78. Data given as means ± SDs for the three independent experiments (* means under *T* test, *p* < 0.05, ** means under *T* test, *p* < 0.01).

**Figure 4:**
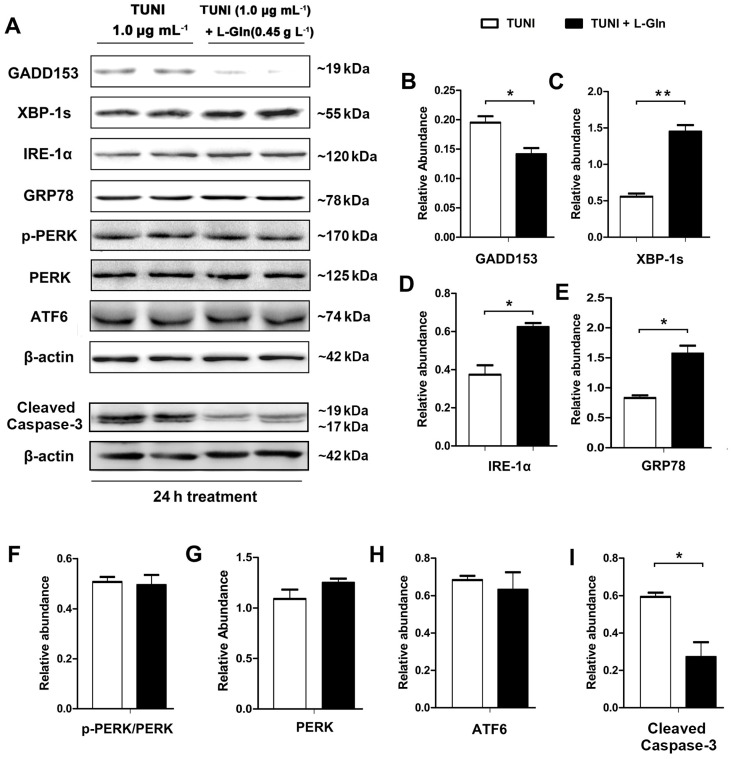
l-glutamine enhances IRE1α-XBP1 axis in ERS-responsive cells. In basic DMEM, cells were treated with 1.0 μg mL^−1^ TUNI or a combination of 0.45 g L^−1^ l-glutamine and TUNI for 24 h. (**A**) Expression of unfolded protein response (UPR) pathway proteins and cleaved caspase-3, respectively, was determined using the Western blot; two representative protein bands from three independent experiments are shown; (**B**) Relative abundance of GADD153; (**C**) Relative abundance of XBP-1s; (**D**) Relative abundance of IRE-1α; (**E**) Relative abundance of GRP78; (**F**) Relative phosphorylation level of protein kinase RNA-like endoplasmic reticulum kinase (PERK) (phospho-PERK (p-PERK)/total PERK); (**G**) Relative abundance of total PERK; (**H**) Relative abundance of ATF6; (**I**) Relative abundance of cleaved caspase-3. β-actin was assessed as a loading control. Data given as means ± SDs for the three independent experiments (* means under *T* test, *p* < 0.05, ** means under *T* test, *p* < 0.01).

To determine whether l-glutamine regulated ERS and ERS pathway mediated apoptosis in the basic DMEM, we treated cells with DMSO, l-glutamine (0.45 g L^−1^) and TUNI (1 μg mL^−1^) or a combination of l-glutamine and TUNI for different durations (0, 8, 16, 24, and 36 h). The results of a cell viability assay using the CCK-8 revealed the survival-promoting effect of l-glutamine in response to TUNI-induced ERS (Figure 3A). This finding was consistent with the results determined by Annexin V-FITC flow cytometry analysis, which showed that ERS-induced apoptosis was attenuated by l-glutamine (Figure 3B,C). To further clarify the anti-apoptotic effect of l-glutamine, we compared GRP78 expression between the TUNI group and the l-glutamine-TUNI group during the initial ERS response (8 h treatment) and the prolonged ERS response (24 h treatment) using immunofluorescence (Figure 3D) and the Western blot (Figure 3E and Figure 4E). We observed that GRP78 expression was not affected by l-glutamine during the initial ERS response, but that l-glutamine maintained a relatively high level of GRP78 during the prolonged ERS response (Figure 3F,G).

### 2.4. l-Glutamine Alleviates C/Enhancer Binding Protein Homologous Protein (CHOP)-Mediated Apoptosis and Activates IRE1α-XBP1 Axis

Next, to explore the mechanisms of the inhibition of cell apoptosis by l-glutamine, we treated the cells with DMSO, l-glutamine (0.45 g L^−1^), and TUNI (1 μg mL^−1^) or a combination of l-glutamine and TUNI for 24 h and performed ERS pathway joint analysis using the Western blot (Figure 4A). As shown, l-glutamine had no effect on the PERK, p-PERK, or ATF-6 pathways in the ERS-responsive cells (Figure 4F–H). However, it downregulated the expression of GADD153/CHOP (Figure 4B) and triggered the upregulation of IRE-1α and XBP-1s (Figure 4C,D). Meanwhile, the expression of cleaved caspase-3, an indicator of cell apoptosis, was reduced by l-glutamine (Figure 4I).

### 2.5. IRE1α-XBP1 Inhibition Reverses Protective Effect of l-Glutamine

**Figure 5:**
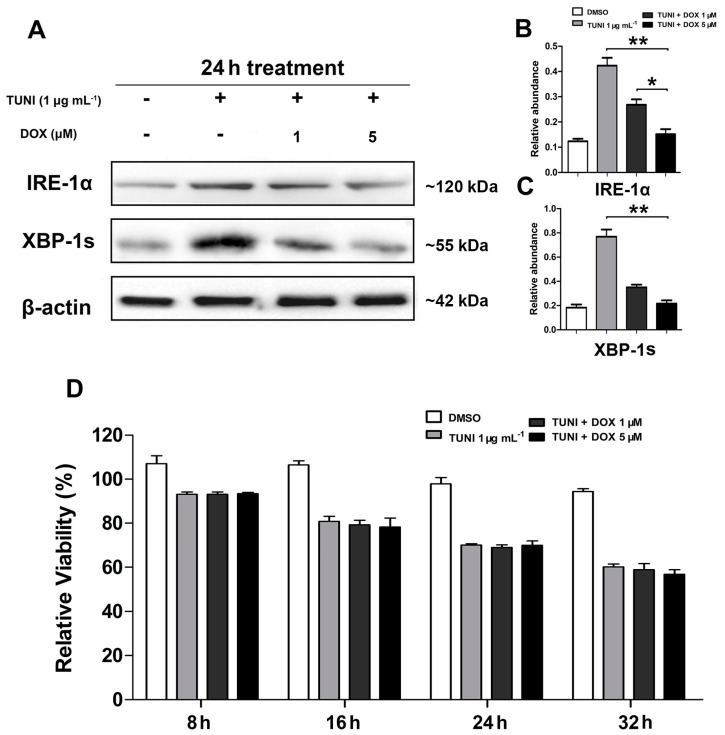
doxorubicin (DOX) inhibits activation of IRE-1α and XBP-1. In basic DMEM, cells were treated with DMSO and 1.0 μg mL^−1^ TUNI or a combination of TUNI and DOX for 24 h. (**A**) Expression of IRE-1α and XBP-1s proteins was determined using the Western blot; one representative protein band from the three independent experiments is shown; (**B**) Relative abundance of IRE-1α; (**C**) Relative abundance of XBP-1s; (**D**) Relative cell viability was measured using CCK-8 after the treatment. β-actin was assessed as a loading control. Data given as means ± SDs for the three independent experiments (* means under *T* test, *p* < 0.05, ** means under *T* test, *p* < 0.01).

**Figure 6:**
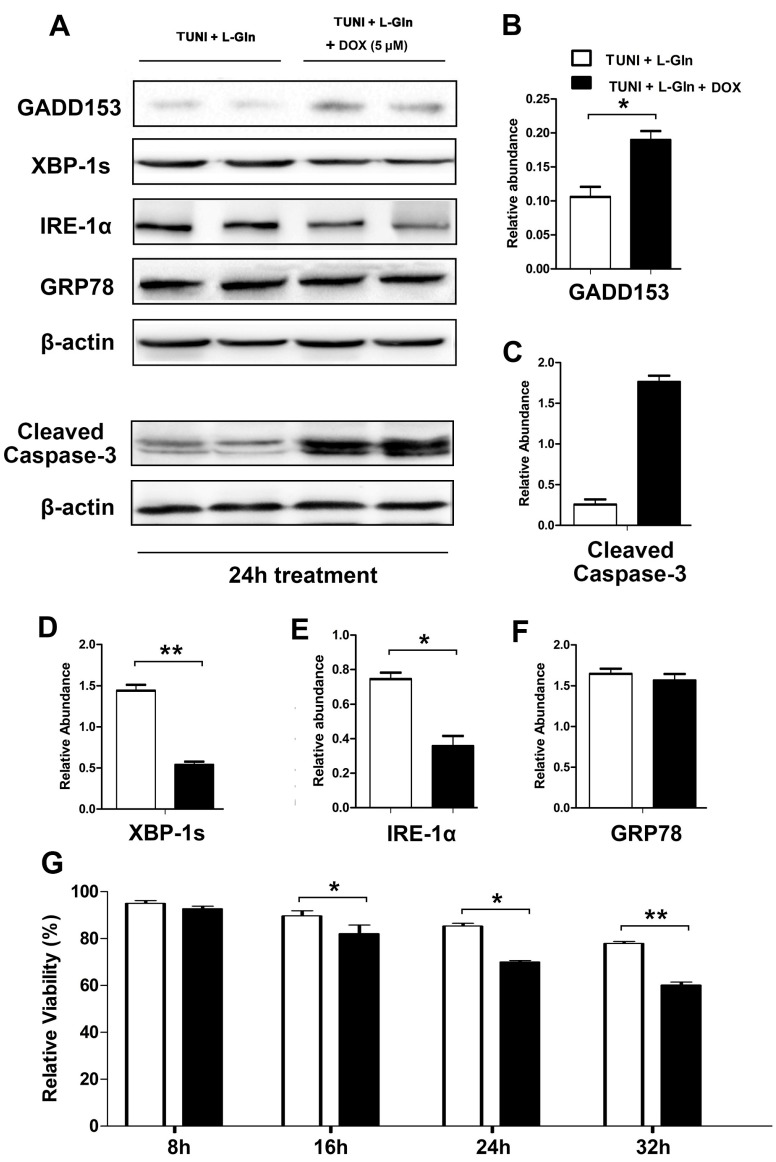
Inhibition of IRE-1α and XBP-1 reverses the anti-apoptotic effect of l-glutamine. In basic DMEM, cells were treated with 1.0 μg mL^−1^ TUNI and 0.45 g L^−1^ l-glutamine or a combination of TUNI, l-glutamine and 4 μM DOX for 24 h. (**A**) Expression of GADD153, IRE-1α, XBP-1s and GRP78 proteins was measured using the Western blot; two representative protein bands from the three independent experiments are shown; (**B**) Relative abundance of GADD153; (**C**) Relative abundance of cleaved caspase-3; (**D**) Relative abundance of XBP-1s; (**E**) Relative abundance of IRE-1α; (**F**) Relative abundance of GRP78; (**G**) Relative cell viability was measured using CCK-8 after treatment. β-actin was assessed as a loading control, and data are given as the means ± SDs for the three independent experiments (* means under *T* test, *p* < 0.05, ** means under *T* test, *p* < 0.01).

To determine whether the l-glutamine-mediated IRE1α-XBP1 axis alleviates ERS-induced apoptosis, we treated IPEC-J2 with doxorubicin (DOX), a specific inhibitor of the IRE1α-XBP1 axis. As shown in Figure 5, DOX inhibited the activation of IRE-1α and XBP-1 in a dose-dependent manner (Figure 5A–C), but had no effect on cell survival (Figure 5D). In addition, 5 μM DOX reversed the anti-apoptotic effect of l-glutamine by blocking the expression of IRE1α and XBP-1s, as shown in Figure 6.

## 3. Discussion

The structure and function of the small intestine in piglets change dramatically on exposure to various stress factors [27]. ERS is considered to be the basis for the progression of many intestinal diseases [21,28], and the health-promoting effect of L-glutamine in the intestine was verified many decades ago [29,30,31], but the precise regulatory mechanisms by which l-glutamine connects ERS with intestinal health are still poorly understood. This study is the first to reveal the role of l-glutamine in mediating ERS-induced cell apoptosis.

TUNI triggers stress by inhibiting the *N*-linked glycosylation of newly synthesised proteins in the ER [32]. This glycosylation controls both the process and the quality of folding [33]. GRP78 is recruited to bind to the unfolded proteins if the hydrophobic parts of the amino acid chain are not successfully buried in the protein interior [34]. Accordingly, researchers have reported that TUNI can induce ERS in various organs in a range of animal species [35,36,37]. However, only rarely have these studies focused on porcine intestinal cells. In the study reported here, TUNI induced the upregulation of GRP78 in IPEC-J2, indicating that ERS had been triggered. The upregulation of ER-colocalised GRP78 has been shown to help cells cope with unfolded proteins during ERS [38]. Notably, prolonged ERS was also found to cause cell apoptosis and GRP78 downregulation, while l-glutamine maintained the level of expression of GRP78 and alleviated the ERS-induced apoptosis, suggesting that the high level of GRP78 maintained by l-glutamine may contribute to its anti-apoptotic effect in ERS-responsive cells. l-glutamine may also affect cell apoptosis by acting as the energy substrate in the body’s response to ERS, thereby maintaining a high level of GRP78. To test this possibility, we replaced l-glutamine with its intermediate product α-ketoglutarate, which plays a functional role in promoting energy metabolism; the AKG replacement did not alleviate the ERS-induced apoptosis (data not shown). Hence, we proposed that l-glutamine may alleviate ERS-induced apoptosis by activating UPR signalling and thereby maintaining the GRP78 level.

The UPR consists of three main signalling branches: PERK, IRE1α, and ATF-6. Prolonged or uncontrolled ERS results in apoptosis [39]. The most important pro-apoptotic pathway in response to ERS has been shown to involve the triggering by PERK of the transcription factor CHOP/GADD153 [40]. Similarly, we found that TUNI treatment increased apoptosis in IPEC-J2 while upregulating GADD153 expression. The response to apoptosis depends on both the condition of the cells and the context of the stress. Our results demonstrated that l-glutamine markedly downregulated the expression of GADD153, suggesting that l-glutamine may decrease PERK pathway mediated apoptosis in ERS-responsive cells. To identify the mechanism of ERS-induced apoptosis, the levels of PERK and p-PERK were determined; however, l-glutamine had little effect on the expression or phosphorylation of PERK. Multiple regulatory modes of GADD153 are likely to exist, due to the downstream genetic activity ofIRE1α and XBP-1s-dependent actions. 

**Figure 7:**
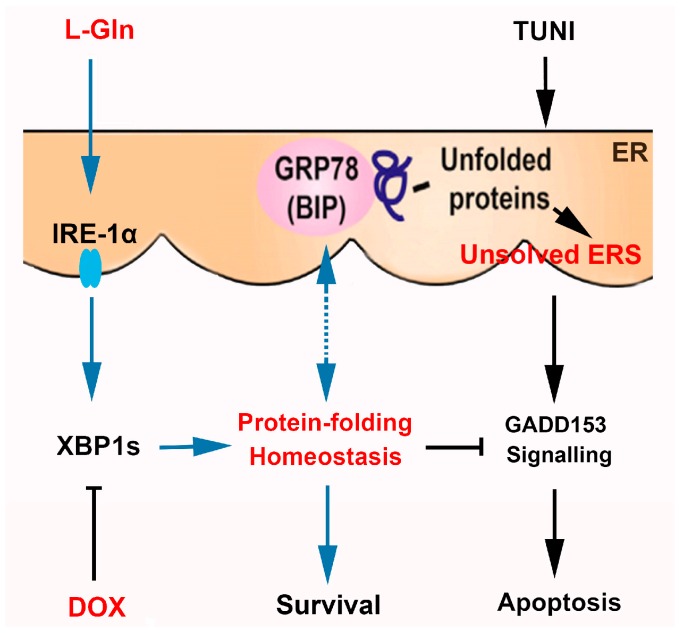
A working model of the role of l-glutamine in protecting an intestinal cell from apoptosis in response to prolonged ERS.

When unfolded proteins accumulate in the ER, IRE1α is activated by autophosphorylation and oligomerisation, triggering RNase activity [28]. As well as inducing XBP1 splicing activity, the activated IRE1 also preferentially degrades ER-related mRNA by cutting at both stem-loop sites and non-stem-loop sites, which is called regulated IRE1 dependent decay (RIDD) [41]. RIDD is made more effective by the silencing of XBP1 splicing [42]. These two processes are thought to reduce the folding load of nascent proteins in the ER and maintain ER homeostasis. Similarly, the results obtained in this study revealed that IRE1α and XBP1s are highly upregulated by l-glutamine in cells responding to prolonged ERS, suggesting that upregulated XBP-1 splicing, not RIDD, is the key signalling mechanism involved in l-glutamine regulated cell survival. To the best of our knowledge, this is the first study to provide evidence of the contribution of l-glutamine to the activation of the IRE1α-XBP1 axis. DOX has been shown to inhibit the IRE1α-XBP1 axis in the HT1080 and RPMI8226 cell lines, and high doses of DOX have been found to threaten cell survival [43]. Consistent with previous reports, the results reported here showed that DOX inhibits the expression of IRE1α-XBP1s in the ERS-responsive cell line IPEC-J2 in a dose-independent manner. However, diverging from previous findings, increasing the concentration of DOX from 1 to 5 µg mL^−1^ was found to have little adverse effect on cell survival within the untriggered ERS milieu. This difference may be due to the cultural milieu used in our study (basic DMEM vs. DMEM with 10% fetal calf serum (FCS) in the relevant previous study) and differences in the sensitivity of cell types to DOX treatment. To identify the anti-apoptotic mechanism of l-glutamine, we optimised the inhibition of XBP-1s by introducing 5 µg mL^−1^ of DOX to inhibit the IRE1α-XBP1 axis. Our results clearly demonstrated that the specific inhibition of IRE1α-XBP1 by 5 µg mL^−1^ of DOX eliminated the anti-apoptotic effect of l-glutamine. This finding strongly suggests that IRE1α-XBP1 upregulation is not a consequence of cell survival but actively helps cells to survive. A working model of the role of l-glutamine in protecting ERS-responsive intestinal cells from apoptosis is provided in Figure 7.

## 4. Materials and Methods

### 4.1. Reagents

Dulbecco’s modified Eagle medium (DMEM), foetal bovine serum (FBS) and antibiotics (penicillin and streptomycin) required for the cell cultures were obtained from GIBCO (Carlsbad, CA, USA). The cell culture plates were manufactured by Corning Inc. (Corning, NY, USA). The CCK-8 and DAPI working solution were purchased from Beyotime Biotechnology (Shanghai, China). l-glutamine, tunicamycin (TUNI), doxorubicin (DOX) and dimethylsulfoxide (DMSO) were purchased from Sigma (Saint Louis, MO, USA). The antibodies against XBP-1s and phospho-PERK were obtained from Santa Cruz Biotechnology, and the antibodies against PERK, ATF6, IRE1α, GRP78, CHOP/GADD153, ATF4 cleaved- caspase3, and β-actin, plus the Alexa Fluor 488-conjugated secondary antibodies, were obtained from Abcam (Cambridge, MA, USA).

### 4.2. Cell Culture and Treatments

The IPEC-J2 cells were grown in DMEM containing 10% FBS, 50 μg mL^−1^ penicillin and 50 μg mL^−1^ streptomycin (a normal growth medium). They were seeded on six-well plates (Corning) at a density of 100,000 cells per well for 36 h. Before the treatments, the cells were washed with phosphate buffered saline (PBS) and the growth culture medium was replaced with basic DMEM without FBS or l-glutamine. First, we treated cells with DMSO, 1 μg mL^−1^ TUNI/DMSO and 0.45 g L^−1^ l-glutamine /DMSO or a TUNI/l-glutamine combination to investigate the molecular mechanism underlying l-glutamine’s regulation of ERS and the effects of this process. The most appropriate concentration of l-glutamine (0.45 g L^−1^) in the treatments was determined in a pre-experiment. Based on the results of our pre-experiment with IRE1α pathway inhibition, we treated cells with DMSO, TUNI/l-glutamine and TUNI/l-glutamine /DOX. We used the DMSO to dilute the TUNI, doxorubicin and l-glutamine, with an equal amount of DMSO added in the control group. We determined through preliminary experiments that exposure to 1.0 μg mL^−1^ TUNI and 5 μM doxorubicin for 24 h represented the most appropriate treatment for the generation of ERS-induced apoptosis and IRE1α pathway inhibition.

### 4.3. Cell Viability Assay

Cell viability was assessed using the CCK-8. The effective component of the CCK-8 is Dojindo’s highly water-soluble tetrazolium salt, WST-8, which can be reduced by dehydrogenase activities in cells to give a yellow formazan dye. The absorbance of each well below 450 nm was taken to indicate the number of living cells. Briefly, cells were seeded in a 96-well plate and incubated with their respective treatments. After a specific duration (0, 8, 16, 24, or 36 h), the cells were incubated with 20 μL CCK-8 for 3 h at 37 °C. Subsequently, the absorbance was read at 450 nm using a microplate reader (Bio-Rad Laboratories, Veenendaal, The Netherlands).

### 4.4. Cell Proliferation Determination by EdU Staining

DNA synthesis during cell proliferation in each group was quantified by incorporating EdU (Invitrogen, Carlsbad, CA, USA) using a Cell-lightTM EdU Kit (Rui Bo Biotechnology Limited Company, Guangzhou, China), as described in our previous studies. Initially, IPEC-J2 cells were cultured in DMEM containing 50 μM EdU for 1 h. A Leica DMI4000B microscope (LEICA, Wetzlar, Germany) was used to capture an image of EdU-positive cells counter-stained with Apollo^®^ 567 fluorochrome (Invitrogen, Carlsbad, CA, USA). The percentage of EdU-positive cells was expressed as the ratio of cells with red nuclei in at least five separate microscopic fields randomly selected for counting.

### 4.5. Flow Cytometry Analysis

Following the treatments, the cells were collected and their apoptotic behaviour was studied by flow cytometry, using an Annexin V-FITC Apoptosis Detection Kit (Beyotime Biotechnology, Shanghai, China) according to the manufacturer’s instructions. Data acquisition and analysis were performed using the flow cytometry system and CELL Quest software (BD Biosciences, Franklin, NJ, USA). Statistical analysis of mortality and apoptosis rates was performed using Prism v5.0 software (GraphPad Software, San Diego, CA, USA) based on the output of the flow cytometry.

### 4.6. Western Blot Analysis

After the treatments, the cells were washed three times in PBS. The resulting samples were lysed for 10 min in an ice-cold buffer with a complete protease inhibitor cocktail, and immunoblotting assays were performed as previously described [44]. The blots were examined using the ECL Plus detection system (Thermo, Waltham, MA, USA) under the conditions recommended by the manufacturer, before signals were visualised using Fujifilm (LAS-3000; Fuji, Tokyo, Japan). The protein band densities were normalised to the specific loading control protein band (β-actin, total PERK) and quantified using Quantity One software (Bio-Rad, Hercules, CA, USA).

### 4.7. Immunofluorescence

The cells were fixed and permeabilised with cold 100% methanol for 10 min on ice. They were then treated with 4 N HCl for 10 min for staining, followed by exposure to 1.5 M Tris-HCl (pH 8.8) for 10 min, before being blocked with 10% goat serum. The cells were incubated overnight at 4 °C with primary anti-GRP78 rabbit polyclonal antibodies. This was followed by incubation for 1 h with Alexa Fluor 488-conjugated secondary antibodies (Life Technologies, Waltham, MA, USA). The nucleuses were incubated with DAPI working solution for 5 min before fluorescence detection. The images were captured using a laser confocal microscope (LEICA, Solms, Germany).

### 4.8. Statistical Analysis

Mean values ± SDs were calculated using Excel 2010 (Microsoft, Seattle, WA, USA) for each of the three independent experiments. The statistical analysis (one-way analysis of variance) was performed using Prism v5.0 software (GraphPad Software, San Diego, CA, USA). This was followed by Bonferroni multiple comparisons and post hoc analysis. Differences between the treatments were considered statistically significant at *p* ≤ 0.01 (**) or *p* ≤ 0.05 (*).

## 5. Conclusions

Our results suggest that l-glutamine protects IPEC-J2 from ERS-induced apoptosis via the IRE1α-XBP1 axis, indicating that the l-glutamine-activated IRE1α-XBP1 axis is crucial to the maintenance of ER homeostasis. These findings provide novel insights into the activity of l-glutamine as a mechanism connecting ERS with intestinal health, and may contribute to a better understanding of intestinal homeostasis.

## Abbreviations

ERS	Endoplasmic reticulum stress
TUNI	Tunicamycin
GRP78	Glucose-regulated protein 78
CHOP	C/enhancer binding protein homologous protein
IPEC-J2	Intestinal porcine epithelial cell line J2
DOX	Doxorubicin
